# Oral health status and its determinants among opiate dependents: a cross-sectional study

**DOI:** 10.1186/s12903-018-0691-3

**Published:** 2019-01-07

**Authors:** Hajar Shekarchizadeh, Mohammad R. Khami, Simin Z. Mohebbi, Hamed Ekhtiari, Jorma I. Virtanen

**Affiliations:** 1 Department of Community Oral Health, School of Dentistry, Islamic Azad University, Isfahan (Khorasgan) Branch, University Blvd, Arqavanieh, Jey Street, P.O.Box: 81595-158, Isfahan, Iran; 2 Research Center for Caries Prevention, Dentistry Research Institute, Tehran University of Medical Sciences, Tehran, Postal code 1417614411 Iran; 3 Community Oral Health Department, School of Dentistry, Tehran University of Medical Sciences, Tehran, Postal code 1439955934 Iran; 4 Neurocognitive Laboratory, Iranian National Center for Addiction Studies, Tehran University of Medical Sciences, Tehran, Postal code 1336616357 Iran; 5 Department of Clinical Dentistry, University of Bergen, N-5020 Bergen, Norway; 6 Medical Research Center, Oulu University Hospital, FI-90029 Oulu, Finland

**Keywords:** Dental caries, Opioid-related disorders, Oral health, Periodontal index, Substance-related disorders

## Abstract

**Background:**

In addition to numerous general health problems, drug dependents manifest various oral health disorders. Our aim was to investigate the oral health status and its determinants among in-treatment opiate dependents.

**Methods:**

As part of a comprehensive cross-sectional survey on opiate dependents admitted to methadone maintenance centers in Tehran, Iran, we conducted a clinical study in two centers from different socioeconomic areas. A trained dentist conducted face to face interviews and clinical oral examinations based on World Health Organization (WHO) criteria for Decayed, Missing, Filled Teeth (DMFT) index and Community Periodontal Index (CPI) on volunteer patients. Student’s t-test, Mann-Whitney U, Kruskal Wallis, and Chi^2^ tests, in addition to linear and logistic regression models served for statistical analysis (*p* < 0.05).

**Results:**

A total of 217 patients (98% men), with a mean age of 43.6 years (SD 12.3) participated in the study. Opium was the main drug of abuse reported by 70% of the participants followed by crystalline heroin (22%). Of the participants, 24.4% were totally edentulous. The mean DMFT score of participants was 20.3 (SD 7.8). Missing teeth comprised the main part of the index followed by decayed and filled teeth. Older patients (*p < 0.001*) and the patients with a lower socioeconomic status (*p = 0.01*) had higher DMFT scores. None of the dentate patients had a healthy periodontium. Maximum CPI mostly consisted of shallow pockets (66%) followed by calculus in 15%, deep pockets in 11%, and bleeding in 8% of the participants. Older participants (*p = 0.02*) and those who started drug abuse at a younger age (*p = 0.01*) were more likely to develop periodontal pockets.

**Conclusions:**

Opiate dependents had a poor oral health status in terms of the dentition status and periodontal health. Missing teeth comprised the main part of their dental caries history and none had a healthy periodontium. Oral health care should be integrated into the package of general health services available in treatment centers.

## Background

Although illicit drug abuse is not as prevalent as cigarette smoking, it can be considered a public health problem in many countries. The public health system bears a heavy burden from drug addiction for prevention and treatment of drug related consequences [1]. Based on the global burden of disease study, 12 million life years are lost due to early death or disability resulted from opioids, cocaine, amphetamines and cannabis, of which about 8 million are linked to opioids [2]. The epidemic of opioids as a continental concern take places via drug trafficking, migration, and borders [3]. Opiates are the most potential harmful opioids with the highest consumption rate and seizures in Southwest Asia [2]. Moreover, North America has encountered the epidemic of opioid misuse, especially in the form of opioid analgesics, heroin, or both [3].

Drug addiction is a preventable condition which has various consequences at an individual level and on the wider society [1, 4]. In addition to several general health problems, a growing number of evidence confirms the high prevalence of oral health problems in people with drug addiction, including generalized dental caries, periodontal disease, mucosal infection, candidosis, mucosal dysplasia, and bruxism [5–8]. These problems might result from the direct effect of drugs on oral health such as the acidic nature of the drugs, drug-induced bruxism, xerostomia, suppression of the immune system, appetite suppression, carving for sweet foods, and blocking the sense of pain. Moreover, they might be associated with drug dependents’ life style factors, including poor nutrition, poor oral hygiene, and alcohol drinking and cigarette smoking in addition to other factors such as low use of dental services and self-medications [8–11].

Health care systems in different countries offer various services for people with drug addiction, including treatment services (medical detoxification, abstinence-oriented treatment, or substitution maintenance therapy), and prevention and harm-reduction facilities (needle-exchange programs, supervised injection facilities, naloxone distribution, and outreach services) [12–14]. Most systems, however, lack comprehensive oral care facilities for drug dependents. Implementation of oral health programs for these patients is challenging due to e.g. addicts’ low priority on their oral health and compliance problems with their treatment plan [4].

Notably, a mutual association exists between addiction and oral health problems. Addiction can exacerbate oral complications on the one hand, and dental pain may result in relapse to drug abuse and reduced addiction treatment success rate on the other hand [15, 16]. Thus, it is crucial to integrate dental care into general health services currently available for people with drug addiction [4]. For planning preventive and curative programs together with general health services for drug dependents, we aimed to investigate the oral health status and its determinants among people with opiate dependence admitted to methadone maintenance treatment.

## Methods

### Subjects

As part of a large cross-sectional survey of people with opiate dependence admitted to methadone maintenance treatment in Tehran, Iran in 2011 [17], we conducted a clinical study in two methadone maintenance programs located in two different socioeconomic areas of the city (north and south). In each center, all patients who met the DSM IV criteria for opioid dependence [18] were asked to participate. Data collection was continued to represent the monthly turnover as most of the patients have monthly visits. This ended to a total of 217 patients.

### Structured interviews about patients’ demographics and drug abuse profile

A trained dental clinician conducted 10-min face-to-face interviews to collect the patients’ demographic characteristics and drug abuse profile. A standard form commonly applied in Iranian addiction studies was used to collect information on the patients’ drug abuse profile (main drug of abuse which made the patient seek treatment, age at the start of drug use, duration of opiate dependence, and duration of current methadone treatment), in addition to their demographic characteristics (age, gender, educational level, marital status, occupational status, and residential area) [17]. As socioeconomic status (SES) indicators, educational level, marital status, occupational status, and residential area were dichotomized as follows: for education: higher education (high school diploma or higher) = 1, lower education (less than high school diploma) = 0; for marital status: married = 1, single (single, widow, divorced) = 0; for employment status: employed (full or part-time) = 1, unemployed (unemployed, retired, housewife, and student) = 0; and for residential area: affluent (north) = 1, non-affluent (south) = 0. The dichotomized scores were then summed up ranging from 0 to 4 [19].

### Oral examinations

The same dental clinician, was trained and calibrated prior to the study. Training and calibration were based on written instructions, and on dental patients. Ten patients were examined and Intra-examiner reliability was more than 0.7. Clinical oral examinations were carried out under the artificial light of a mobile dental unit. DMFT (Decayed, Missing, and Filled Teeth) and CPI (Community Periodontal Index) using CPI probes and disposable dental mirrors were recorded according to the WHO criteria [20]. Criteria of decayed teeth were clinically visible lesions (cavity or undermined enamel), or when the explorer tip penetrated to the soft structures. No tooth cleaning was undertaken before the examination.

### Statistical analysis

Statistical Package for the Social Science (SPSS for Windows, version 16.0/PC; SPSS, Chicago, IL, USA) was used for data analysis. Mean DMFT and corresponded Standard Deviation (SD) were calculated. Student’s t-test, Mann-Whitney U, Kruskal Wallis, and Chi^2^ tests were employed for statistical analysis (level of significance < 0.05). Linear and logistic regression models were fitted to the data to analyze the factors associated with drug dependents’ DMFT and periodontal disease, respectively.

## Results

### Demographics and drug abuse profile

As high as 98% of the participants were men (only 5 women). The mean age of the patients was 43.6 years (SD 12.3, rang 21–79). Table 1 presents the demographic characteristics of in-treatment opiate dependents. The majority of the patients had more than primary school education (87%), were married (73%), and were employed (68%).

**Table 1:** Demographic characteristics of opiate dependents in methadone maintenance treatment (*n* = 217)

Variable	Number	Percentage
Gender (n, %)		
Male	212	98
Female	5	2
Age (n, %)		
18–24	9	4
25–34	48	22
35–44	62	29
45–54	50	23
55–64	39	18
65≤	9	4
Educational status (n, %)		
Illiterate	7	3
Elementary school	21	10
High school	145	67
University	43	20
Marital status (n, %)		
Married	158	73
Single	35	16
Widow	4	2
Divorced	20	9
Employment status (n, %)		
Full time job	104	48
Part time job	44	20
Unemployed	32	15
Student	1	0.5
Retired	30	14
Housewife	5	2

Opium was the main drug of abuse reported by 70% of drug dependents followed by crystalline heroin (22%). The mean age of opium dependents (47 yrs) was higher than that of crystalline heroin users (35 yrs) ((*p < 0.001*). The mean age at the start of drug use was 25.2 years (SD 8.8; range 11–67), and the mean duration of opiate dependence was 12.1 years (SD 8.6). The mean duration of current methadone treatment was 10.0 months (SD 15.3) (Table 2).

**Table 2:** Drug abuse profile of opiate dependents in methadone maintenance treatment (*n* = 217)

Variable	Number	Percentage
Main drug of abuse		
Opium	152	70
Crystalline heroin	48	22.1
Other drugs	17	7.8
Age at start of drug abuse		
≤ 17	31	14.4
18–24	84	38.9
25–34	71	32.9
35 ≤	30	13.9
Duration of opiate dependence (year)		
< 1	5	2.3
1–5	53	24.8
6–10	62	29.0
11 ≤	94	43.9
Duration of current methadone treatment (month)		
< 1	30	13.9
1–5.9	84	38.9
6–11.9	50	23.1
12–23.9	24	11.1
24 ≤	28	13.0

### Oral health status

About one quarter (24.4%) of the participants (*n* = 53) were totally edentulous. The mean DMFT of opiate dependents was 20.3 (SD 7.8; range 0–28). Missing teeth comprised the main part of the index (mean 13.6; SD 10.3; range 0–28) followed by DT (mean 4.8; SD 4.8; range 0–20) and FT (mean 1.8; SD 3.1; range 0–16). Table 3 presents the mean DMFT of the participants according to their background characteristics and drug abuse profile. In univariate analysis, a higher DMFT score was associated with older age (*p < 0.001*). Single participants and those who were employed showed lower DMFT scores (*p < 0.001*). The mean DMFT of opium dependents (21.7; SD 6.9) was higher than that of crystalline heroin dependents (16; SD 8.3) (*p < 0.001*). Patients who started drug use at an older age and those who had a longer history of drug dependence showed higher scores of DMFT (*p < 0.001*).

**Table 3:** DMFT scores of in-treatment opiate dependents according to their backgrounds and drug abuse profile (*n* = 217)

Variable	DMFT (Mean, SD)	*p*-value
Gender		
Male	20.3 (7.7)	0.852*
Female	18.4 (11.5)	
Age		
18–24	8.2 (4.9)	< 0.001**
25–34	14.6 (7.9)	
35–44	20.1 (6.5)	
45–54	22.5 (6.0)	
55 ≤	26.2 (4.2)	
Education		
0–5	22.2 (7.5)	0.302**
6–12	20.1 (7.7)	
13 ≤	19.4 (8.3)	
Marital status		
Married	21.1 (7.2)	0.001**
Single	15.3 (9.0)	
Others	22.1 (7.2)	
Employment status		
Full-time job	18.5 (7.8)	< 0.001**
Part-time job	18.8 (8.3)	
Unemployed	22.7 (6.2)	
Others (student, retired, homemaker)	24.9 (6.6)	
Area of residence		
Affluent	20.1 (7.8)	0.594*
Non-affluent	20.8 (7.9)	
Main drug of abuse		
Opium	21.7 (6.9)	< 0.001**
Crystalline heroin	16.0 (8.3)	
Other drugs	19.2 (9.7)	
Age at start of drug abuse		
≤ 17	14.7 (8.3)	< 0.001**
18–24	19.8 (7.7)	
25–34	21.4 (7.1)	
35 ≤	24.3 (5.7)	
Duration of opiate dependence (year)		
< 1	11.4 (11.2)	<.001**
1–5	17.2 (8.0)	
6–10	19.4 (7.6)	
11 ≤	23.1 (6.4)	
Duration of current methadone treatment (month)		
< 1	19.7 (7.4)	0.870**
1–5.9	19.5 (8.9)	
6–11.9	20.7 (6.8)	
12–23.9	21.6 (5.0)	
24 ≤	20.9 (8.4)	

* Mann-Whitney U, ** Kruskal Wallis Test

In multivariate analysis, older drug dependents (*p < 0.001*) and patients with a lower socioeconomic status (*p = 0.01*) had higher scores of DMFT (Table 4).

**Table 4:** Factors associated with DMFT of in-treatment opiate dependents as shown by a linear regression model

Varriable	All patients (210)^c^			Dentate patients (162)^d^		
	Beta Coefficient	*P*-value	95% CI	Beta Coefficient	*P*-value	95% CI
Age	0.48	< 0.001	0.18–0.43	0.49	< 0.001	0.24–0.43
Socioeconomic status^a^	−0.14	0.016	−1.98-(−0.21)	−0.16	0.016	−2.11-(−0.18)
Main drug of abuse (Crystalline heroin)^b^						
Opium	0.12	0.077	−0.22-4.31	0.07	0.383	−1.31-3.39
Others	0.04	0.514	−2.38-4.75	0.04	0.567	− 2.81-5.11
Age at start of drug abuse	0.01	0.912	−0.14-0.15	− 0.07	0.470	− 0.25-0.17
Duration of opiate dependence (year)	0.11	0.164	−0.04-0.24	0.12	0.145	−0.04-0.28
Duration of current methadone treatment (month)	0.07	0.221	−0.02-0.09	0.08	0.258	−0.04-0.15

^a^Combination of the following variables: area of residence, education, marital and employment status

^b^Reference group

^c^R^2^ = 0.40

^d^R^2^ = 0.25

Among dentate participants, the mean DMFT was 17.8 (SD 7.4), which mostly consisted of missing teeth (mean 9.0; SD 7.2). The mean DT and FT was 6.4 (SD 4.6) and 2.4 (SD 3.4), respectively. Similar results were found in multivariate analysis of factors associated with DMFT among dentate opiate dependents (Table 4).

None of the dentate patients had a healthy periodontium. Max CPI mostly consisted of shallow pockets (66%) followed by calculus (15%), deep pockets (11%), and bleeding (8%). In univariate analysis, periodontal disease (pocket formation) was associated with none of the background or drug abuse variables (*p > 0.05*). Table 5 reveals factors associated with pocket formation among dentate opiate dependents. Older participants (*p = 0.02*) and those who started drug abuse at a younger age (*p = 0.01*) were more likely to develop periodontal pockets.

**Table 5:** Factors associated with periodontal disease (pocket formation) among dentate dependents by logistic regression model (*n* = 148)

Variable	ES	SE^d^	*P*-value	OR	95% CI
Age	0.07	0.03	0.017	1.07	1.01–1.13
Socioeconomic status^b^	0.01	0.22	0.98	1.01	0.67–1.52
Main drug of abuse (Crystalline heroin)^c^					
Opium	− 0.09	0.50	0.86	0.92	0.35–2.43
Others	−0.10	0.74	0.89	0.90	0.21–3.82
Age at start of drug abuse	−0.09	0.04	0.014	0.92	0.86–0.98
Duration of opiate dependence	0.02	0.05	0.65	1.02	0.93–1.11
Duration of current methadone treatment (month)	−0.03	0.02	0.066	0.97	0.93–1.00

^a^male = 0, female = 1

^b^Combination of the following variables: area of residence, education, marital and employment status

^c^Reference group

Goodness of fit with Hosmer and Lemeshow test (*p* = 0.66)

^d^Standard Error

## Discussion

The present study of oral health and its determinants among opiate dependents revealed poor oral health in terms of dentition and periodontal health. Missing teeth comprised the main part of their dental caries history and none of them had a healthy periodontium. Older drug users and patients of lower socioeconomic status were at greater risk of developing oral problems.

### Drugs and health / health behavior / oral health

Patients with drug addiction show numerous health consequences, including heart attack, respiratory depression, liver cirrhosis, nephropathy, and infectious diseases such as hepatitis, AIDS, and tuberculosis [4]. In addition, mental disorders such as depression, injury-associated disabilities, and oral health problems are common in drug dependents [4].

In addition to the direct effects of drugs on the oral health, unhealthy behaviors are common in drug dependents. General personal neglect and poor dental hygiene, irregular eating pattern, high intake of carbohydrates, tobacco and alcohol dependence, low dental attendance, and low priority for oral health are lifestyle factors contributing to their oral health problems [4, 21].

### Dental caries experience

The mean DMFT of the patients was 20.3 which mostly consisted of missing teeth (mean 13.6). The mean DMFT of opiate dependents in our study was about twice as high as the mean DMFT of the general middle-aged population in Tehran (mean: 11) [22]. However, MT comprises the main part of the index among both drug dependents and the general population. Compared to the general population (3%) [22], about 16% of our study participants were totally edentulous. The highest DMFT score (26.2) was seen in the oldest age group (55 ≤) while the youngest (18–24) had the lowest score (8.2) probably due to effect of age on dentition.

The DMFT of a group of in-treatment Spanish drug dependents was 22.7, excluding the four edentulous patients from analysis. Similar to our study, missing teeth comprised the main part of the index [5]. The mean DMFT of a group of drug dependents undergoing rehabilitation in Brazil (mean 14.9) [23], a group of remand prisoners in London (mean 14.2) [24], and a community of in-treatment alcohol and/or drug dependent persons from 3 outpatient public clinics in Brazil (13.0) [25] was lower than the mean DMFT in our study. The mean DMFT of a group of heroin users treated with methadone in a Chinese city [26] and a group of illicit drug dependents in a rehabilitation center in India [27] was 3.9 and 3.5 respectively, which is much lower than what we found. In both abovementioned studies, the prevalence of dental caries in their general populations was also lower than that of the Iranian general population [26, 27]. The lower DMFT score of the Chinese and Indian drug dependents might also be the result of the high percentage of people who were totally edentulous in our study, or might be due to the higher mean age of our participants (40.5 yrs) compared to the other two studies (about 35 yrs).

### Periodontal health status

None of the patients in our study had a healthy periodontium. Similar results were found in Chinese heroin users (0%) and Indian drug dependents (2%) [26, 27]. In the present study (66%) and in Indian drug dependents (44%) [27], the max CPI index mostly consisted of shallow periodontal pockets. However, in the Chinese study, calculus comprised the main part of the index (66.3%), followed by shallow periodontal pockets (27.6%) [26].

Similar to our study, healthy periodontium was rare in middle aged general population of Tehran (1%), and shallow periodontal pockets (43%) and calculus (40%) comprised the main parts of the index [22]. Based on another study in India, compared to the general population, drug dependents showed higher periodontal destruction and lower gingival bleeding [28]. Plaque and periodontal diseases were also prevalent among remand prisoners in London [24]. It seems that periodontal diseases are more prevalent among drug dependents in Iran than other countries [26, 27]. The prevalence of periodontal diseases is high in both Iranian opiate dependents and the general population. However, the higher percentage of periodontal pockets as an indicator of periodontal destruction among drug dependents points out an urgent need to establish oral health promotion programs for people with drug addiction.

### Background and drug abuse profile

In our study, the mean DMFT of opium dependents was higher than that of crystalline heroin dependents. This seems to be a result of the higher mean age of opium dependents than that of crystalline heroin users in our study (47 vs. 35 years respectively), which is consistent with other studies from Iran [17]. It is also commonly known that the DMFT index is higher among older age groups. Patients with a lower socioeconomic status showed higher DMFT scores. However, among Chinese former heroin users, DFT scores were related to sex and the duration of drug abuse after controlling the effect of education, occupation and age [26]. Among drug dependents in Australia, age, gender, and dose and/or duration of drug abuse were associated with dental destruction [29].

Regarding periodontal health, older dependents and those who started drug abuse at a younger age were more likely to develop periodontal pockets. Similar findings were reported in former drug dependents in China. Periodontal health was associated with duration of drug abuse and age of the participants [26]. Based on the study by Gupta et al. in India, drug abuse was independently related to a poor periodontal status [27].

### Limitations and strengths of the study

Considering the scarcity of the data about oral problems among people with drug addiction, we managed portray oral health and its determinants in Iranian opiate dependents. However, no casual interpretation is possible due to the cross-sectional nature of the study. On the other hand, we studied opiate dependents attending maintenance treatment programs; thus, the results cannot be generalized to all drug dependents including prisoners and other street drug users. Objective data collection via clinical examination based on the WHO criteria and using a mobile dental unit provided valid information about participants’ oral health status. In addition, the patients’ demographics and drug abuse profile were collected via individual interviews instead of commonly applied self-administered questionnaires to increase the validity of the data. Recruiting patients from treatment centers in two main different socioeconomic areas of the city of Tehran is another strong point of the study.

## Conclusion

The present study revealed a poor oral health status among opiate dependents in terms of their dentition status and periodontal health. Missing teeth comprised the main part of their dental caries history and none of them had a healthy periodontium. Older drug dependents and patients of lower socioeconomic status were at greater risk of developing dental problems. Thus, it is recommended to integrate oral health care into the package of general health services available in treatment centers. Educational, preventive, and treatment programs focusing on oral health should be targeted to these high risk patients in rehabilitation settings.

## Abbreviations

CPI: Community Periodontal Index
DMFT: Decayed, Missing, Filled Teeth
SD: Standard Deviation
WHO: World Health Organization
